# Community perceptions on outdoor malaria transmission in Kilombero Valley, Southern Tanzania

**DOI:** 10.1186/s12936-017-1924-7

**Published:** 2017-07-04

**Authors:** Irene R. Moshi, Halfan Ngowo, Angel Dillip, Daniel Msellemu, Edith P. Madumla, Fredros O. Okumu, Maureen Coetzee, Ladslaus L. Mnyone, Lenore Manderson

**Affiliations:** 1 Department of Environmental Health and Ecological Sciences, Ifakara Health Institute, Dar-Es-Salaam, United Republic of Tanzania; 2 School of Public Health, Faculty of Health Sciences, University of the Witwatersrand, Parktown, Johannesburg, Republic of South Africa; 3 Wits Research Institute for Malaria, School of Pathology, Faculty of Health Sciences, University of the Witwatersrand, Sandringham, Johannesburg, Republic of South Africa

**Keywords:** Community knowledge, Malaria prevention, Outdoor malaria transmission, Rural Tanzania

## Abstract

**Background:**

The extensive use of indoor residual spraying (IRS) and insecticide-treated nets (ITNs) in Africa has contributed to a significant reduction in malaria transmission. Even so, residual malaria transmission persists in many regions, partly driven by mosquitoes that bite people outdoors. In areas where *Anopheles gambiae* s.s. is a dominant vector, most interventions target the reduction of indoor transmission. The increased use of ITNs/LLINs and IRS has led to the decline of this species. As a result, less dominant vectors such as *Anopheles funestus* and *Anopheles arabiensis,* both also originally indoor vectors but are increasingly biting outdoors, contribute more to residual malaria transmission. The study reports the investigated community perceptions on malaria and their implications of this for ongoing outdoor malaria transmission and malaria control efforts.

**Methods:**

This was a qualitative study conducted in two rural villages and two peri-urban areas located in Kilombero Valley in south-eastern Tanzania. 40 semi-structured in-depth interviews and 8 focus group discussions were conducted with men and women who had children under the age of five. The Interviews and discussions focused on (1) community knowledge of malaria transmission, and (2) the role of such knowledge on outdoor malaria transmission as a contributing factor to residual malaria transmission.

**Results:**

The use of bed nets for malaria prevention has been stressed in a number of campaigns and malaria prevention programmes. Most people interviewed believe that there is outdoor malaria transmission since they use interventions while indoors, but they are unaware of changing mosquito host-seeking behaviour. Participants pointed out that they were frequently bitten by mosquitoes during the evening when outdoors, compared to when they were indoors. Most participants stay outdoors in the early evening to undertake domestic tasks that cannot be conducted indoors. House structure, poor ventilation and warm weather conditions were reported to be the main reasons for staying outdoors during the evening. Participants reported wearing long sleeved clothes, fanning and slapping themselves, using repellents, and burning cow dung and neem tree leaves to chase away mosquitoes.

**Conclusions:**

Community understanding of multiple prevention strategies is crucial given changes in mosquito host seeking behaviour and the increased incidence of outdoor biting. The current low use of outdoor control measures is attributed largely to limited awareness of outdoor transmission. Improved community understanding of outdoor malaria transmission is critical: efforts to reduce or eliminate malaria transmission will not be successful if the control of outdoor transmission is not emphasized.

## Background

The burden of malaria has decreased significantly in many endemic countries, particularly in African countries where over 90% of the cases and deaths worldwide are recorded. In some sub-Saharan countries, including in North Africa and West Africa (including Algeria, Gambia, Senegal and Guinea-Bissau) malaria cases and deaths have decreased by over 72%. Similar trends were observed in East Africa between 2000 and 2015, with a 75% decrease in case incidence in Madagascar, Rwanda, Tanzania and Ethiopia [1]. Despite this achievement, malaria remains the major health problem globally, responsible for approximately 212 million cases and 429,000 deaths worldwide [2].

Children under the age of five and pregnant women are particularly vulnerable to malaria because of low or suppressed levels of immunity [3]. Globally, about 303,000 malaria related deaths occur in children under 5 years of age [2]. Reflecting this vulnerability, the main focus of malaria control in Tanzania and elsewhere has been on mosquito control interventions, timely and effective case management, and intermittent treatment in pregnant women [4]. However, the control of mosquito vectors, mainly using long lasting insecticide-treated nets (LLINS) and indoor residual spraying (IRS), is the mainstay of malaria control and has consequently led/contributed to the significant reductions in the incidences of malaria.

In Tanzania, the National Malaria Control Programme (NMCP), in collaboration with other stakeholders such as intervention manufacturers, distributors, funders, research and religious institutions, has adopted a series of initiatives to enhance the uptake and sustained use of malaria control interventions. In the 1990s, social marketing was introduced in Kilombero Valley to promote use of bed nets, with the distribution of subsidized bed nets through the KINET project under the slogan “ZuiaMbu” [5–7]. This was followed by the distribution of large numbers of bed nets designed to cover all sleeping places [5], an approach that was piloted by NMCP in a few regions in Tanzania and subsequently extended nationally [8, 9]. These initiatives led to the increased uptake of bed nets, reduction of human–mosquito interaction, increased protection from bites, and a reduction of malaria. Other measures consolidated these gains: proper diagnosis and treatment, house improvements such as house screening, covering all the eaves with ceilings [10, 11], and use of personal repellents.

Despite these advances in malaria control, efforts are hindered by emerging strains of insecticide resistance and the inability of the frontline vector control measures to target outdoor biting mosquitoes [1, 12–23]. Current frontline vector control measures, LLINs and IRS, exclusively target indoor biting mosquitoes. Recent studies indicate an increasing risk of outdoor malaria transmission due to changes in mosquito behaviour from indoor (endophagic) to outdoor (exophagic) biting [21–25]. There is evidence that up to 50% of transmission now occurs outdoors in some localities in Kilombero Valley in Tanzania and Nyanza in Kenya [20, 26]. This may vary between rural and urban settings for a number of reasons, including geographical location, the economic status of householders influencing how house environs are used, living arrangements, and the presence of other social amenities. The few available outdoor vector control interventions, including larval source management, topical repellents and treated clothes. However these interventions are yet to be scaled up, leaving the majority of residents in rural communities with insufficient knowledge and awareness of their use to control outdoor malaria transmission.

The recurrence of malaria has been observed in areas where LLINs and other control interventions are widely used. The reasons for recurrence are unknown but human behaviour appears to play a fundamental role in such instances [27]. Several human behaviours appear to increase the risk of disease exposure and malaria transmission. These include sleeping outdoors during hot weather [23] and all night ceremonies that contribute to exposure to mosquitoes [28]. When farming and during the harvesting season, farmers within Kilombero Valley spend considerable time living on farmland (*shamba* in Swahili) in dwellings made from branches and straw, and spend most of their time outdoors [29]. In this area where malaria transmission is experienced almost throughout the year, understanding human behaviours in various environments that increase the risk of malaria is critical for developing complementary outdoor based vector control interventions [30]. The study we describe here explored peoples’ understanding of malaria transmission in Kilombero Valley, focusing on knowledge of outdoor malaria transmission, human practices that increase vulnerability to outdoor transmission, and protective practices that people presently use to avoid being bitten when outdoors.

## Methods

### Study area

The study was conducted in Ulanga and Kilombero districts, Kilombero Valley, south-eastern Tanzania, from June to July 2014 and February to March 2016. The study was conducted in two villages in Ulanga, a predominantly rural district, and two villages in Kilombero district, a more urbanized area. The total area of Kilombero Valley, which lies at approximately 300 m above sea level, is 73,039 km^2^, the equivalent to 8.2% of the total area of the Tanzania mainland [31]. The annual rainfall ranges from 1200 to 1800 mm, with the rainy season normally running from December to May, and the dry season from June to November. The annual temperature ranges from 20 to 32.60 °C. The main economic activities in the area are rice and maize cultivation, and fishing in Kilombero River. Malaria prevalence in Kilombero Valley is 14% [32]. The estimated entomological inoculation rate (EIR) was above 300 infectious bites per person per year in the 1990s [33], but has since decreased to 9.19 as estimated from a CDC light trap catches and 15.87 as estimated from HLC catches, and both are highly contributed by An.funestus. The area is characterized by intense and persistent malaria transmission from *Plasmodium falciparum,* peaking during the rainy season [34, 35] even though malaria transmission continues throughout most of the year.

### Study design

A purposive study design was used in the selection of households, as explained in the next section. Data were collected with qualitative methods including in-depth interviews (IDIs) and focus group discussions (FGDs). The study was guided by grounded theory principles, including following a system of analytic steps which generate social theories and concepts to describe events and situations [36]. The approach was iterative, insofar as data collected were immediately analysed to see if the questions were understood and to allow us to reflect on the findings, so informing the modification of the tools for subsequent data collection.

### Selection of study participants

Purposive sampling was used to select households across the four study villages which are estimated to have a total of 7478 households. The inclusion criteria were (1) the household that are near the main road and at the edges of the villages for logistic purposes, (2) the household should have at least one child aged 0–5 years. Of 176 households which met the inclusion criteria only 128 households were available, out of which 40 households were selected, 10 from each of the four study villages. In-depth interviews were conducted with one member of the selected households, either one of the parents or any other household member above 18 years of age. The interviews were complemented by focus group discussions, two from each study village to make a total of eight FGDs. Each group comprised an equal number of men and women. FGD participants were selected in collaboration with village leaders. Each FGD had participants ranging from 10 to 12. All participants who participated in the study gave out their consent and signed the consent forms.

### Data collection

The IDIs were conducted between June and July 2014; and were supplemented with FGDs conducted between February and March 2016. The IDIs were conducted at the participants’ households while FGDs were conducted at the village offices or vacant classrooms at nearby schools, in venues that were accessible and convenient for study participants. The IDIs and FDGs guides were developed, piloted and revised before starting the actual data collection. A member of the research team obtained informed consent from individuals before we conducted the IDIs and FGDs. Both interviews and focus groups discussions were conducted in Kiswahili language, and were tape-recorded for a complete record and for quality assurance. The recordings were complemented by shorthand notes.

### Data processing and analysis

All audio data were transcribed, translated and double checked for clarity prior to data processing and analysis. Thematic content analysis was conducted based on the principle concepts and themes, by ordering, structuring and interpreting the collected data. The themes and subthemes identified included knowledge of malaria transmission, perceptions of outdoor malaria transmission, reasons for outdoor activities and use of control measures. The qualitative data analysis computer software package (Nvivo software version 13) was used to arrange the rich text-based information of the transcribed interviews.

## Results

### Characteristics of study participants

The IDI participants comprised of 20 males and 20 females whose ages ranged from 18 to 79 years. The majority of participants had received basic primary school education; a few had secondary or tertiary education. The FGD participants comprised of 45 male and 43 female caregivers/household heads whose ages ranged from 21 to 65. More than half of the FGDs participants had basic primary education, few had secondary education and very few had reached tertiary education. All participants for both IDIs and FGDs were subsistence farmers regardless of place of residence, education and income level. Their economic status was determined based on the properties they owned, for example a house, land, car, motorcycle, bicycle, television, radio, and cell phone. The economic status of IDI participants included 25% low income, 57.5% middle income and 17.5% high income farmers. The economic status of FGD participants included 22% low income, 60% middle income and 18% high income farmers.

### Perceptions on outdoor malaria transmission

The majority of study participants in both IDIs and FGDs still believed that malaria transmission occurred disproportionately indoors, and were surprised when they were still infected with malaria. Because of this experience, they speculated that on-going malaria transmission was linked to outdoor biting, since they experience high numbers of bites between 6 and 8 p.m. They reported that they had been bitten more often outdoors than indoors, with a high level of malaria episodes even though bed net coverage and use was high. They also considered that transmission might occur both indoors and outdoors.

“*We normally use mosquito bed nets while inside the house, but we still get malaria. It might be that the mosquito bites we get when outdoors give us malaria*”

(Middle income subsistence farmer, IDI)

“*We are bitten by mosquitoes while outdoors, since we do not have any means of protection, they bite us and infect us with malaria*”

(Middle income subsistence farmer, FGD)

Mosquito biting rates were relatively high during the rainy season, and study participants were aware that this was associated with increased number of mosquito breeding sites as a result of standing rain water.

“*We are bitten more by mosquitoes during the rainy season. For instance, when it rains there are so many puddles which are mosquitoes breeding sites, as well as in the bushes since there are many bushes at that time and they are their preferred areas*”

(Middle income subsistence farmer, FGD)

### Time spent outdoors

Most participants indicated that they spend longer times outdoors than indoors in the early evening to perform different activities, and are most often bitten by mosquitoes whist outdoors. The most common activities leading people across the study villages to spend long periods outdoors include cooking, eating outdoors (dinner), washing kitchen utensils, and participating in conversation with other family members. Spending a long time outdoors during the night was largely dictated by the source of energy used for food preparation, as well as the lack of sitting and kitchen space indoors. The outdoor activities were mainly done by women and children. Nevertheless, the adult men were also exposed to outdoor bites as they often sat idle while waiting for dinner or spent time at a bar or kiosk away from home, again primarily sitting outdoors where it is relatively cool. During summer, when indoor heat become extremely uncomfortable, people spend much longer periods outdoors than they do in other seasons where most people go inside the houses to sleep between 8 and 9 p.m.

“*Mosquitoes bite us more when we are outdoors during the evening from around 7* *p.m. because that is the time we are most outdoors while cooking. Maybe you are cooking late, mosquitoes bite you but when it reaches 9* *p.m., you go inside the house and go to sleep under the net*”.

(Middle income subsistence farmer, FGD)

“*Times like these, which is summer, it is too hot inside, so we must sit outside and get fresh air until midnight then go inside to sleep*”.

(Middle income subsistence farmer, FGD)

### Malaria control measures and reasons for use

The majority (>95%) of participants indicated that bed nets were the most common and widely used indoor mosquito control intervention in their communities. In rare cases, people used indoor insecticide sprays, while topical repellents, wearing long sleeved clothes, slapping one self, and burning cow dung and leaves from neem trees (*Azadirachta indica*) were the main interventions used as protection from mosquito bites when outdoors.

Overall, the use or non-use of interventions in the study communities was driven by affordability and acceptability of an intervention as well as fear for sickness, hospitalization, death, and mosquito nuisance.

“*Malaria disturbs me a lot. This disease is my enemy*”.

 (Low income subsistence farmer, IDI),

“*It is a very dangerous disease that affects/infects women and children*”.

 (High income agricultural farmer, IDI)

“*I do not want to get sick, because I will die*”.

 (Middle income subsistence farmer, IDI)

### Sources of information

Information regarding malaria transmission and prevention plays a crucial role in the way that people behave towards disease exposure and prevention. The information on malaria transmission with which most study participants were aware focused on most likely period of being bitten by mosquitoes and getting malaria, and the use of bed nets as the main malaria control intervention. The majority perceived that they could only get malaria if bitten by mosquitoes indoors. The participants further indicated that they received this and other malaria related information through one or several of the following means: radio, television, at school, program implementers and researchers. Moreover, the participants maintained that communications did not portray the increasing risk of malaria transmission, variation in time and place, nor its associated socio-cultural risk factors.

“*I got the information from the media, television and radio, and from the people who come to educate us* (researchers/health programme implementers).* They tell us how to protect ourselves and the products to use for protection such as repellents,* (and)* treat our mosquito bed nets*”.

 (Low income subsistence farmer, IDI)

### Perceptions on malaria prevention and treatment

There is a confusion regarding ongoing malaria transmission within the study villages and the Valley, despite the high use of available interventions, such as insecticide-treated nets (ITNs/LLINs). People use these interventions, but still get fevers and so seek medical care so they feel that the disease is far from disappearing. The use of bed nets is seen to be the only protection from malaria since many programmes have stressed this intervention, but they use leaves from neem tree, cow dung and long sleeve clothes to chase away mosquitoes. Other interventions such as indoor residual spraying with insecticides (IRS), mosquito coils and larval source management (LSM) were rarely mentioned by the respondents from both FGDs and IDIs. Furthermore, people avoid the use of other interventions such as topical repellent because of assumed negative health effects.

“*I heard that such products cause skin cancer that is why I never want to use it, I better fan myself*.”

 (Middle income subsistence farmer, IDI)

“*We use bed nets when inside the house. While outside, I once used repellent, but I stopped because I am allergic to it*.”

 (Middle income subsistence farmer, IDI)

The study participants revealed that despite people’s awareness of malaria and its transmission, individualistic behaviour hinders prevention of the disease. People specifically spoke of the decline of spirit of togetherness which was built since the development of social and economic policy by the Late Julius Kambarage Nyerere, the former President of Tanzania soon after Independence which focused on collective agriculture under a process called villagilization—‘family hood’ which was popularly known as “UJAMAA” [37]. Contrasting a time in the past when everyone was responsible for the protection of people with the present, a modern world in which individualism has become more pronounced. In the past, participants explained, all community members would feel concerned and voluntarily assume responsibility for whatever was happening in and around their respective communities. One respondent suggested that individualism was taking over the sense of togetherness, leading to misunderstandings and hindering the prevention of malaria transmission.

“*Even if you find a child of your neighbour outside, you cannot force them to go inside as a way of protecting them from mosquito bites outside or punish them when they refuse to do so because you might cause problems with the parents. It is different these days, unlike way back when your neighbours’ child was perceived as yours even if it was your neighbour’s, but now things have changed, you cannot touch someone else’s child*.”

 (High income subsistence farmer, FGD)

Participants were also suspicious of the efficacy of antimalarial drugs. Many spoke of how they completed a course of medication yet still contract the disease, suggesting the potential for re-infection of drug resistant strains. Artemisinin-based combination therapy (ACT) (popularly known as artemether–lumefantrine, i.e. ALU) is viewed by a minority of participants as a prescribed drug which does not cure malaria. Villagers prefer previous common anti-malarial drugs, such as chloroquine, and proposed it be returned to the market because it did not require multiple doses as is the case with the current medication:

“*Chloroquine used to be the best anti*-*malarial drug, unlike this available drug of nowadays which does not cure, so they should just bring it back*”.

 (Middle income subsistence farmer, IDI).

## Discussion

The risk of malaria transmission and performance of its control interventions are strongly influenced by human behaviours that determine exposure to mosquito bites. Therefore, deep understanding of these behaviours is critical for developing sustainable complementary outdoor based vector control interventions. The problem of outdoor transmission, increasing in many endemic countries including Tanzania, is seriously compromising global malaria elimination efforts. The study investigated peoples’ perceptions on malaria and their implications to ongoing outdoor malaria transmission and malaria control efforts.

There are concerns that many people are still contracting malaria despite the wide use of LLINs and that people are more often bitten outdoors than indoors imply on-going outdoor malaria transmissions in the study area. This is in line with other studies where minimal protection against malaria was apparent even under full coverage with LLINs and IRS [38–41]. Night-time outdoor activities, in a setting where the malaria vectors exhibit at least some exophagic and exophilic behaviour, increase the risk of outdoor biting and malaria transmission [42]. The most common night activities reported within the study area included cooking, washing kitchen utensils, and conversation among family members. Furthermore, children and adult men often sit idle while waiting for dinner, or in the case of men, at a bar or a kiosk away from home, again sitting outdoors. These activities are habitual, thus indicating long-standing risk of exposure to outdoor mosquito bites and malaria transmission in the area, more so because nearly 99% of the local vector population is comprised of *Anopheles arabiensis* which is strongly exophagic and exophilic [21, 43]. The night time activities comparable to those observed in this study have been described elsewhere [23, 44, 45]

Children under the age of five and pregnant women are more vulnerable to malaria. We envision considerable consequences of their exposure to mosquito bites outdoors when pregnant women and other women conduct household chores during the evening accompanied by children as observed in other parts of Africa [23, 45]. Outdoor control interventions need to be prioritized due to the increased outdoor biting and the close proximity of households to breeding areas. Furthermore, people in the study areas rely primarily on the use of bed nets indoors to prevent malaria [28], and so remain unprotected before going to bed and/or when outdoors. During summer, the situation is worse as people spend more time outdoors because of the heat [38].

The study participants claimed rare use of protection outdoors, including topical repellents, wearing long sleeved clothes, slapping one self, and burning cow dung and leaves from neem tree (*A. indica*), as also reported in Ghana [23]. This is a motivation for scaling up and advocating promising outdoor-based interventions such as spatial repellents and larval source management (LSM) [25, 46, 47]. In scaling up, there is a need to ensure that these and other interventions are rendered acceptable, affordable and accessible to rural and other poor communities. The study participants described affordability and accessibility as the main determinants for use or non-use of malaria control interventions.

To enhance adoption of outdoor malaria control interventions, advocacy on changing patterns of malaria transmission needs to go hand in hand with the scale up. In contrast to the previous malaria awareness campaigns, we must ensure communities receive up to date and reliable information about malaria trends. In the current study, it was identified that the study communities still relied on outdated messages, most of which were misconceived and misleading. Notable examples of such messages include *Zuia Mbu*—*Malaria huambukizwa usiku wamanane* (protect yourself from mosquitoes; malaria is unacceptable) distributed in 1996 and 1997 through community sensitization and written on sachets distributed with bed nets [7, 48, 49], and *Malaria Haikubaliki, Tumia chandarua* (malaria is unacceptable, use bed nets), disseminated by Population Service International (PSI) from 2010 to 2015. These messages date from the 1990s and early 2000s, when mosquito bites and malaria transmission were predominantly occurring indoors. No efforts have been made to revise them. The current understanding of outdoor host-seeking behaviour needs to be packaged well and communicated to target communities, preferentially using multiple forms of communication, such as radios, televisions, and posters to heighten community awareness on the upcoming challenges in disease control.

The timely sharing of information on interventions and changes in host seeking behaviour will reduce misconceptions and hearsay on interventions and could influence uptake and compliance of existing vector control interventions and anti-malarial drugs. The majority of malaria transmissions, particularly in sub-Saharan Africa, still occur indoors. Therefore, there is a need to ensure that messages are well packaged so that people continue to use LLINs, IRS, drugs and other recommended interventions. Wrong messages on drug efficacy may have serious consequences, particularly when there is limited choice in terms of the outdoor based interventions that poor rural communities can afford and access. In the current study, some of the participants complained that ALU was not effective against malaria. This may be the result of a few people failing to adhere with the prescribed dose and therefore becoming re-infected with malaria parasites [50]. In Ghana, factors such as individuals’ blood type and spiritual interventions were believed to be causing malaria treatment failure [51], but also there were negative perceptions associated with taking and using ACT for malaria treatment [52]. Information needs to be communicated constructively to communities, to prevent people’s resistance to these drugs and to support on-going efforts to combat outdoor malaria.

## Conclusions

Outdoor activities, mainly domestic chores, expose people to mosquito bites. The existing education on intervention use which focuses on bed net use, as per advertisements and programme implementation messages, will not reduce existing outdoor biting exposure. Campaigns which incorporate education on existing transmission outdoors, outdoor interventions, and their accessibility are important. Control strategies which target outdoor biting mosquitoes and complement those interventions that work indoors are necessary for progress towards elimination. Nevertheless, the global community needs to explore the possibilities of reducing the cost of these interventions to encourage their adoption, while developing other options. Accessibility of outdoor interventions and use will complement the existing indoor interventions, reducing the rate of residual transmission and contributing to disease prevention.

## Abbreviations

ACT: artemisinin-based combination therapy
ALU: artemether–lumefantrine
EIR: entomological Inoculation Rate
IDI: in-depth Interview
IHI: Ifakara Health Institute
IRB: Institutional Review Board
IRS: insecticide residual spray
ITN: insecticide-treated net
LLIN: long-lasting insecticidal net
NMCP: National Malaria Control Programme
MRCC-NIMR: Medical Research Coordinating Committee at the National Institute for Medical Research in Tanzania
